# Vasectomy re-reversal: effectiveness and parameters associated with its success

**DOI:** 10.1590/S1677-5538.IBJU.2020.0310

**Published:** 2020-12-20

**Authors:** Mariana S. Lorenzini, Fernando Lorenzini, Cícero A. Bezerra

**Affiliations:** 1 Hospital de Los Angeles Departamento de Urologia CuritibaPR Brasil Departamento de Urologia, Hospital de Los Angeles, Curitiba, PR, Brasil

**Keywords:** Spermatozoa, Vasectomy, Vasovasostomy

## Abstract

**Introduction:**

When the vasectomy reversal (VR) fails, and the patient desires natural conception with his sperm, vasectomy re-reversal (VRR) is the only alternative.

**Purpose:**

To determine the VRR effectiveness and whether specific parameters can be associated with its success.

**Materials and Methods:**

We retrospectively evaluated 18 consecutive vasectomized patients, who had failed their VR through bilateral vasovasostomy, and posteriorly were submitted to VRR. The parameters of the study were: age of the patients, elapsed time between vasectomy and VRR (V-VRRt), elapsed time between VR and VRR (VR-VRRt), presence of spermatozoa in the proximal vas deferens fluid (SptzVDF) in the VRR and results of semen analysis after VRR (SA-VRR).

**Results:**

The mean of the age of the patients was 44.11±6.55 years (32.0-57.0), the mean of V-VRRt was 11.76±6.46 years (1.5-25.0) and the mean of VR-VRRt was 2.13±2.27 years (0.5-10.0). SptzVDF in the VRR were found bilaterally in 8 patients, unilaterally in 4 and absent in 6. SA-VRR demonstrated normozoospermia in 9 patients, oligozoospermia in 3 and azoospermia in 6, with patency rate of 66.67%. SA-VRR showed statistically significant dependence only with SptzVDF in the VRR (p <0.01).

**Conclusions:**

VRR was effective in restoring the obstruction in more than half of the patients. Furthermore, the presence of spermatozoa in the vas deferens fluid was the parameter associated with the VRR success.

## INTRODUCTION

Vasectomy has been widely used as a surgical contraception. In the United States, it was estimated that 175.000 to 354.000 vasectomies have been performed annually between 1998 and 2002 (1), however around 4 to 6% of the vasectomized patients will still desire to father children with their spermatozoa (2), that can be treated by the vasectomy reversal (VR), in vitro fertilization or intracytoplasmic sperm injection of the oocytes with spermatozoa retrieved from the patient (3, 4).

The VR failure is defined as a persistent postoperative azoospermia, or a very good sperm concentration per mL with very small or even without motility and/or associated with necrozoospermia. In these cases, and when the patient desires natural conception with his own sperm, the vasectomy re-reversal (VRR) is the only alternative for conceiving a child, although a repeat microsurgery may be considered as a challenge for the urologist (5).

There are few articles on the VRR, most of which are dated from the last decades (2, 5-8). Therefore, there is a need for more research, especially using specific parameters to be associated with its results. The hypotheses of this study are that VRR could be effective in a significant number of patients and at least one specific parameter could be associated with its success, helping them to decide on this repeat microsurgery.

In a consecutive vasectomized patients, who had failed their VR through bilateral vasovasostomy and posteriorly were submitted to VRR, the objectives of this study were to determine the VRR effectiveness and whether any specific parameter could be associated with its success.

## MATERIALS AND METHODS

This article retrospectively evaluated 18 consecutive vasectomized patients who had failed their VR through bilateral vasovasostomy (VV), posteriorly were submitted to VRR and wanted the natural conception with their own sperm. The follow-up period was 12 months or more after the VRR. This study was approved on the Institutional Board according to the ethical requirements for scientific publication (OF. 001.5/20).

The parameters of the study were: age of the patients, the time elapsed between vasectomy and VRR (V-VRRt), the time elapsed between VR and VRR (VR-VRRt), the presence of spermatozoa in the proximal vas deferens fluid (SptzVDF) in the VRR and the semen analysis after VRR (SA-VRR). The 2010 WHO laboratory manual was the one used for semen analysis evaluation and categorizing the patients as normozoospermic or otherwise (9).

In order to determine the SptzVFD in the VRR, the fluid from the lumen of the proximal vas deferens was collected by direct aspiration and freshly analyzed under optical microscopy. Whether spermatozoa were present in this fluid, or when there were no such spermatozoa, but this fluid had a clear appearance and flowed continuously from the vas deferens, the technique for the reconstitution of the seminal pathway would be the VV in a single plane with 9-0 nylon stitches, otherwise the reconstruction would be the end-lateral microsurgical vasoepididimostomy (VE) in a single plane with 10-0 nylon stitches.

### Statistical Analysis

All the parameters were analyzed in relation to SA-VRR, employing ANOVA test if W test of Shapiro-Wilk and K^2^ test of Bartlett showed normality and homogeneity of the variance of the parameters, respectively, otherwise Kruskal-Wallis’ chi-square test (X^2^) was used. Pearson’s chi-square test (X^2^), Fisher’s exact test and Cramer’s V test were used to verify the independence of the parameters with SA-VRR.

It was adopted the confidence level greater than 99% and statistical difference of less than 1% (p <0.01).

## RESULTS

All the data and the results of the parameters of the study are summarized in Tables 1 and 2.

**Table 1:** Data and results of the parameters of the study.

Patient No.	Patient age (years)	V-VRRt (months)	VR-VRRt (months)	SptzVDF VR	SptzVDF VRR	SA-VRR Seminal volume / Sperm concentration / Progressive motility / Normal sptz forms
1	32	48	9	ni	0	2.3mL / Absence of spermatozoa
2	42	300	12	ni	2	3.2mL / 55 x 10^6^sptz/mL / 35% / 4%
3	39	96	11	ni	1	1.6mL / 54 x 10^6^sptz/mL / 40% / 6%
4	47	120	36	ni	2	5.5mL / 25 x 10^6^sptz/mL / 35% / 4%
5	40	132	18	1	2	2.1mL / 1 x10^6^sptz/mL / < 1% / ni
6	57	288	120	ni	0	1.7mL / Absence of spermatozoa
7	43	96	6	0	0	3.8mL / Absence of spermatozoa
8	42	192	12	2	2	2.7mL / 27 x 10^6^sptz/mL / 40% / 6%
9	48	228	19	2	2	1.5mL / 146 x 10^6^sptz/mL / 32% / 5%
10	53	96	8	ni	0	2.8mL / Absence of spermatozoa
11	44	135	24	0	0	3.1mL / Absence of spermatozoa
12	45	192	54	2	2	3.6mL / 34 x 10^6^sptz/mL / 35% / 4%
13	43	96	48	ni	1	1.9mL / 70 x 10^6^sptz/mL / 33% / 5%
14	49	108	17	ni	2	2.5mL / 24 x 10^6^sptz/mL / 44% / 6%
15	53	18	15	ni	1	2.2mL / 0.01 x 10^6^sptz/mL / 0% / ni
16	44	156	15	ni	1	2.1mL / 11 x 10^6^sptz/mL / 28% / 2%
17	41	181	26	2	2	2.0mL / 61 x 10^6^sptz/mL / 37% / 6%
18	32	57	9	ni	0	1.5mL/ / Absence of spermatozoa

**Sptz** = spermatozoa; **V-VRRt** = time elapsed between vasectomy and vasectomy re-reversal (VRR); **VR-VRRt** = time elapsed between vasectomy reversal (VR) and VRR; **SptzVDF VR and SptzVDF VRR** = presence of sptz in the vas deferens fluid in the VR and in the VRR, respectively, as follow, **ni** = no information (patients from another urological centers); **0** = no sptz was found; **1** = sptz were found in only one of the vas deferens; **2**: sptz were found in bilateral vas deferens; **SA-VRR** = semen analysis after VRR.

**Table 2:** The presence of spermatozoa in the vas deferens fluid in the vasectomy re-reversal and the semen analysis after this surgery.

SptzVDF VRR	Semen analysis after the VRR (number of patients)		
	Azoospermia	Oligozoospermia	Normozoospermia
0	6	0	0
1	0	2	2
2	0	1	7

**Sptz** = spermatozoa; **VRR** = vasectomy re-reversal; **SptzVDF VRR** = presence of sptz in the vas deferens fluid in the VRR as follow; **0** = no sptz was found; **1** = sptz were found in only one of the vas deferens; **2** = sptz were found in bilateral vas deferens.

The mean age of the patients was 44.11±6.55 years (32.0 to 57.0), the mean of V-VRRt was 11.76±6.46 years (1.5 to 25.0), and the mean of VR-VRRt was 2.13±2.27 years (0.5 to 10.0).

SptzVDF in the VRR were found bilaterally in 8 patients (44.44%), unilaterally in 4 (22.22%) and absent in 6 (33.33%). The vas deferens fluids in all VRR demonstrated spermatozoa, or when there were no such spermatozoa, these fluids had a clear appearance and flowed continuously from the vas deferens, indicating the absence of epididymal obstruction.

SA-VRR showed normozoospermia in 9 patients (50.00%), oligozoospermia in 3 patients (16.67%) and azoospermia in 6 patients (33.33%), with a patency rate of 66.67%.

The VR-VRRt was the only one parameter that satisfied the normality and the homogeneity of the variance, in relation to the SA-VRR (lowest value of normality was W=0.801, p <0.060; highest value of homogeneity of variance was K^2^(2)=0.884, p <0.642), allowing to use ANOVA test.

The age of the patients was not associated with SptzVDF in VRR (X^2^(2)=0.003, p <0.998), nor with SA-VRR (X^2^(2)=0.090, p <0.956). V-VRRt did not show any statistical significant difference with SptzVDF in VRR (F(2, 15)=2.517; p <0.114) nor with SA-VRR (F(2, 15)=1.175; p <0.336). VR-VRRt did not show any statistical significant difference with SptzVDF in VRR (X^2^(2)=2.628; p <0.269), nor with SA-VRR (X^2^(2)=2.569; p <0.277). In other words, there is no statistical relation between the age of the patients, V-VRRt and VR-VRRt with the SptzVDF or with SA-VRR.

The SptzVDF in VRR and SA-VRR, using Chi-square, Cramer’s V and Fisher’s exact tests, showed that there was no statistically significant independence between these two parameters (X^2^(4)=21, p <0.000; V=0.764, Fisher p <0.000). As there was a statistically significant dependence only between SptzVDF and SA-VRR, SptzVDF was the parameter associated with the VRR success.

## DISCUSSION

VRR means to repeat a microsurgery that has already been performed but, for this purpose, the knowledge of its effectiveness and the parameters associated with its success would help the patient to make his decision on this procedure.

Belker et al. (2) reported that VRR had lower results than VR and, in a total of 222 VRR performed before the year 1991, spermatozoa were found in the SA-VRR in 75% of them, however there was no consensus among the parameters of VRR success.

Fox (5), studying 22 patients who had failed their VR, concluded that the cause of the obstruction of the seminal pathway, in the majority of the cases, occurred at the anastomotic site and, employing the two-layer VV, obtained spermatozoa in the ejaculate in 64% of them. On the other hand, Silber (10) suggested that, after a long time from vasectomy and using an accurate VV, the obstruction could be secondary to the rupture of epididymal tubules.

Hernandez et al. (6), analyzing 33 patients submitted to VRR, 79% of them resulted in patency and, according to the smoking history, obstructive interval from vasectomy, type of reconstruction (VV on at least one side versus VE alone) and conception, only the last one was associated with pregnancy.

Paick et al. (7) reported 62 VRR, 58 of them underwent bilateral VV, two only one unilateral VV and two unilateral VV with contralateral VE, suggesting that VV should be performed when surgically possible. The overall patency and pregnancy rates were 92% and 57%, respectively. The increased age of the wives was a negative prognostic factor for pregnancy (p <0.018). They also found that the compromised anastomosis is the most common cause of failed VR and SptzVDF, obstructive interval, reconstruction type, anastomotic site, patient age and SA-VRR did not influence the VRR outcome.

In the present study, VRR was considered an effective microsurgery, showing normozoospermia and oligozoospermia in 50.00% and 16.67% of the patients, respectively, with a patency rate of 66.67%. Analyzing the parameters age of the patients, VRRt, VR-VRRt, and SptzVDF in relation to SA-VRR, only SptzVDF was the parameter associated with the VRR success. The pregnancy was not adopted as a criterion of VRR outcome, since it involves variables related to the different causes of female infertility that may compromise this rate. All VRR were performed through VV, that can be explained by the fact that all VR were also done using VV and, besides this, all the fluids of the proximal vas deferens demonstrated spermatozoa, or when there were no such spermatozoa, these fluids had a clear appearance and flowed continuously from the lumen of the vas deferens, considered as favorable fluids, allowing to employ VV reconstruction. In six cases of azoospermia, which were coincident to the parameter SptzVDF without spermatozoa, VV anastomoses were done due to the presence of favorable fluids in all of them and, analyzing retrospectively this scenario, we believe that the patients may have had a compromised spermatogenesis or an obstruction after VRR that determined these results of the SA-VRR. Otherwise, if any patient would have had no spermatozoa in the analysis of SptzVDF in the VRR, with unfavorable or absent fluids, the seminal tract would have been reconstructed trough VE anastomosis.

Another important aspect on VRR is that, even if the patient had a failed VR and posteriorly underwent the percutaneous epididymal sperm aspiration, it is still possible to perform the VRR, supported by the reports that SptzVDF were found in VR after a long time from this sperm recovery, indicating the absence of epididymal obstruction (11, 12).

Finally, the limitations of this study were a relatively small, but a meaningful number of patients submitted to a repeated microsurgery and, therefore, more parameters could not be studied. We intend to increase the number of VRR in order to find more associations between the parameters and the prognosis.

## CONCLUSIONS

The VRR was an effective microsurgery, restoring the obstruction in more than half of the patients. Furthermore, only SptzVRR was the parameter associated with the VRR success.
